# Exploring public discourses about emerging technologies through statistical clustering of open-ended survey questions

**DOI:** 10.1177/0963662512441569

**Published:** 2013-10

**Authors:** Paul Stoneman, Patrick Sturgis, Nick Allum

**Affiliations:** University of Southampton, UK; University of Essex, UK

**Keywords:** biotechnology, discourses of science, public understanding of science

## Abstract

The primary method by which social scientists describe public opinion about science and technology is to present frequencies from fixed response survey questions and to use multivariate statistical models to predict where different groups stand with regard to perceptions of risk and benefit. Such an approach requires measures of individual preference which can be aligned numerically in an ordinal or, preferably, a continuous manner along an underlying evaluative dimension – generally the standard 5- or 7-point attitude question. The key concern motivating the present paper is that, due to the low salience and “difficult” nature of science for members of the general public, it may not be sensible to require respondents to choose from amongst a small and predefined set of evaluative response categories. Here, we pursue a different methodological approach: the analysis of textual responses to “open-ended” questions, in which respondents are asked to state, in their own words, what they understand by the term “DNA.” To this textual data we apply the statistical clustering procedures encoded in the Alceste software package to detect and classify underlying discourse and narrative structures. We then examine the extent to which the classifications, thus derived, can aid our understanding of how the public develop and use “everyday” images of, and talk about, biomedicine to structure their evaluations of emerging technologies.

## 1. Introduction

A persistent dilemma within science governance is the question of how to deal with the democratic demands placed upon regulatory and legislative institutions when technologies are being rapidly developed and applied, and the public and policy makers struggle to keep pace with their uses, implications, risks, and benefits (Ruckelshaus, 1986; Kleinman, 2000; Salter and Jones, 2006). Historically, science governance has been a policy domain driven almost entirely by “elites,” with little opportunity for the preferences of the general public, such as they are, to feed into funding and regulatory decision-making. In response to this apparent “democratic deficit,” a range of public engagement and consultation procedures have been established over the past ten to fifteen years, with the goal of enabling ordinary citizens to have more influence on the direction of science policy and practice (Salter and Jones, 2002; Frewer and Salter, 2007). These include citizens’ juries, such as the NanoJury UK (Rogers-Hayden and Pidgeon, 2006), consensus conferences (Joss and Durant, 1995), deliberative mapping (Burgess et al., 2007), and focus groups (Holliman, 2005) which all aim, in one way or another, to bring ordinary members of the general public into closer engagement with the technical, social, and ethical issues around new and emerging science and technology. While such strategies have all met with varying degrees of success, they nonetheless face the charge that, being based on small and self-selecting samples, they do not adequately reflect the true distribution of views and preferences within the public as a whole (see Holliman, 2005: 257–258; Middendorf and Busch, 1997; Rowe and Frewer, 2000: 12–13).

Although not generally conceived of as being part of the apparatus of public engagement, a crucial link between science and the public – and one which has considerably stronger claims to represent the full distribution of public opinion – is social surveys and opinion polls. These are the key conduit through which public opinion and preferences regarding the speed and direction of policy, including that relating to science and technology, are presently communicated to governmental and non-governmental institutions and stakeholders (Wlezien, 2005; Althaus, 2008). It is also, of course, through the findings of opinion polls and attitude surveys that the media often intervene to report on public fears about, or hostility towards, an area of scientific practice, often in the form of a headline grabbing majority rejecting a particular technology or idea for future application. The motivation underlying this paper, however, is the oft-cited observation that standard “closed-format” survey questions are not ideal instruments for delineating complex, dynamic, and potentially “un-formed” preferences about science and technology within the general public (Irwin and Wynne, 1996; Sturgis and Allum, 2004; Nisbet and Goidel, 2007). This is because, when a technology is unfamiliar and cognitively demanding to understand, from both a technical and an ethical perspective, it seems unlikely that survey designers will be capable of pre-determining the full range of responses that might be given by members of the public about it, when asked. Under such conditions, it is certainly possible that the standard closed-format survey question does not so much reveal pre-existing public opinion about the technology in question, as create it, a critique that has long dogged the social survey more generally (Moscovici, 1963; Converse, 1964; Bishop, 2004).

In recognising that closed-ended questions may constrain or distort our understanding of public responses to new scientific issues and technologies, we explore in this paper whether quantitative analysis of unstructured, verbatim responses to “open-ended” survey questions can provide a solution to the problems associated with measuring public opinion about techno-science via a narrow and pre-determined set of fixed alternatives. To be sure, the approach we apply here – so-called “*quantitizing*” of qualitative data (Sandelowski, Voils and Knafl, 2009) – is just one way of combining qualitative and quantitative methods, and one that might, indeed, stand accused of merely transforming the qualitative into the quantitative as opposed to being a genuinely *integrative* approach. Be that as it may, our goal here is not to resolve such definitional issues of method but to explore, pragmatically, whether this particular approach might be a useful tool for the analysis of public understandings of, and reactions to, new and emerging areas of science. The remainder of the paper is set out as follows. First, we describe the social and political context in which the elicitation of public opinion about science and technology is situated, before reviewing some of the methodological challenges that arise when asking questions about low-salience and cognitively demanding societal issues. We then describe the data and key measures upon which our analysis is based and present our key results. We conclude with a discussion of the substantive implications of our findings and an evaluation of the methodology employed.

## 2. Science policy and public opinion

The nature and direction of public opinion is a key battleground for political elites and commentators on a wide range of policy issues, with the domain of science and technology being no exception. The primary reason for this is simple: it is the manifestation of a struggle for legitimacy. Where the appropriate course of action is uncertain and contested, principles of representative democracy mean that having “the public on your side” can provide decisive momentum in debates over the speed and direction of policy (Dahl, 1989). As recent time-series evidence has shown, governments appear to be responsive to short and long run movements in prominent public opinion polls relating to the relative priorities for government spending – the so-called “thermostatic model” of the relationship between public opinion and policy making (Wlezien, 2005; Soroka and Wlezien, 2011).

Yet, in affording the notion of public opinion a normative role in the formulation of policy, particularly between elections, how “public opinion” is measured and interpreted becomes not just a technical scientific challenge but also a question of democratic legitimacy. For, in treating public opinion as coterminous with what is measured by opinion polls, there is a real danger that the “will of the people” might easily be misrepresented as a result of technical shortcomings or deliberate malpractice by vested interests who wish to push for a particular legislative or regulatory position (Fishkin and Luskin, 2005). In short, the idea that policy makers are responsive to opinion polls is comforting only insofar as polls and surveys can be taken as accurately reflecting the “true” state of public opinion. However, there are numerous examples from the empirical record that should give us pause for thought before accepting the idea that opinion polls are an unproblematic way of measuring the pre-formed attitudes residing in the heads of survey respondents. To name but a few prominent examples, survey respondents have been shown to willingly offer opinions on non-existent issues (Bennett, 1975; Bishop, Hamilton and McConahay, 1980; Sturgis and Smith, 2010); to switch from one side to the other of prominent issues in a quasi-random manner over time (Converse, 1964; Iyengar, 1973; Asher, 1974; Sturgis, 2002); and to provide very different answers depending on the way in which questions are administered to them (Schuman and Presser, 1981).

In the area of science policy, these reservations have been evident in recent controversies about biotechnology. The GM Nation? debate in the UK in 2003, for example, found that 86% of the public were against eating genetically modified food. This figure received high profile media coverage but was out of line with contemporaneous high quality survey evidence (Sturgis et al., 2004), a discrepancy likely to reflect the self-selecting nature of the sample design and the ability of lobby groups to deliberately over-represent themselves in the achieved sample (Pidgeon et al., 2005). In 2008, following the creation of “part-human, part-animal” embryos, the *Daily Mail* newspaper reported that “two out of three people are against the creation of hybrid embryos,”^1^ a figure generated from a survey commissioned by the Catholic Church which asked respondents whether they “support or oppose allowing scientists to create embryos which are part-human part animal.” However, according to independent surveys, which posed the question in a less value-laden manner, less than half of the British public were opposed to this practice (Jones, 2009: 169).

## 3. Closed-ended questions and non-attitudes

In the examples described above, “closed-format” measures of public opinion from surveys and polls played a key role in shaping the public debate on science policy. Respondents to these surveys were asked to express their position by selecting one of a limited number of pre-specified evaluative descriptors relating to a statement about the technology in question, such as “agree” or “strongly agree.” The limitations of the random sample survey for uncovering the complexities and dynamism of public opinion are well known (Blumer, 1969; Herbst, 1992), and our central concern here is that, in constraining the range of opinions available to be expressed to a predefined set of answers that are themselves selected by the researcher (or funder), we obtain a representation of public opinion which is “rigged” in advance to reflect the (often implicit) assumptions of those who commissioned and designed the survey (Mayer and Stirling, 2004).

In addition to the potential for closed-ended questions to shape or steer responses in a particular direction, a further consideration is that even when people have little or no understanding of the science they are being asked to evaluate, many will select one of the fixed alternatives offered to them rather than admit ignorance, simply because the formalities and conventions of the survey interview stipulate that providing answers is “what you are supposed to do” (Converse, 1964; Bishop et al., 1980). These types of “non-attitudes,” or “pseudo-opinions” can represent a large proportion of all responses on questions about issues of scientific complexity and are, unsurprisingly, more prevalent amongst the less scientifically knowledgeable members of the public (Sturgis and Smith, 2010). For these reasons, it seems sensible to ask whether close-ended questions are the most appropriate way of understanding public opinion about complex areas of science and technology, particularly if the results are intended to feed into policy and regulatory decision-making.

## 4. The potential value of open-ended questions

An obvious alternative to presenting a set of fixed response alternatives is to ask respondents to report their thoughts and perspectives on a particular issue in their own words and for interviewers to record these responses “verbatim.” The potential advantage of this type of “open-ended” question is that it allows the respondent to use his or her own frame of reference in determining a response, even if this might seem inappropriate or “irrational” to the survey designer or analyst. Thus, this approach should result in a fuller and more heterogeneous set of perspectives than the standard closed-format question. Additionally, of course, the amount of information about an individual’s position on an issue that can be derived from an open question is considerably greater than that which is afforded by a closed-ended alternative. And, indeed, many large scale academic surveys have employed open-ended questions, particularly in the pioneering period of survey research in the United States, during the post-war era (Converse, 1987). Most notable in this regard is the American National Election Survey series which has fielded open-ended questions on American political issues since the 1940s and which formed the basis of the important idea that the American public can be stratified into different “levels” of ideological sophistication, based on the content of responses to these questions (Converse, 1964).

Despite their appeal as a means of avoiding the shortcomings of fixed response alternative questions, open-ended questions are not widely used in survey research today. One reason for this is that concerns have long been raised about whether open-ended questions favour the articulate and well-educated, who are likely to provide longer and potentially richer responses and, thereby, exert a disproportionate influence on public policy (see Sturgis and Allum, 2006; though see Greer, 1988 for a counter-position). On a more practical level, open-ended questions are not used frequently due to the high cost of fielding these questions because they take longer for interviewers to administer and require additional resources to transcribe and to code into a frame. Even when open-ended questions are included in a survey, it is rare for analysts to use them in a way that exploits the richness of the additional information provided by the full-text strings. Instead, they are generally employed by analysts in a quantitative manner, as if the question had been asked in a closed-ended format in the first place, raising further questions about the returns for the additional cost of including them. It is clear that, partly, this tendency is a result of “habitual practices”; quantitative researchers using the procedures which they are comfortable and familiar with. It is also, however, due to the fact that there are few well-established procedures for using verbatim responses in any other way, beyond their occasional use as quotations to offer a “flavour” of the kinds of things people said, alongside a conventional quantitative analysis. What is needed, then, is a more systematic and robust methodology for the analysis of open-ended survey questions that is capable of utilising the rich semantic information of the verbatim responses, but in a way that retains the possibility of reliable population inference.

A response to this methodological challenge has been the development of “Computer Assisted Text Analysis” (Brier and Hopp, 2010) otherwise known as CAQDAS (“Computer Assisted Qualitative Data Analysis Software”) approaches (Fielding and Cisneros-Puebla, 2009). Moving beyond mere word counts, these approaches and their associated software utilise quantitative techniques such as multidimensional scaling (MDS) and correspondence analysis (CA) to identify structures within a piece of text based on the co-occurrence of words (see Murtagh, 2005 for an extensive and technical review of the latter). This enables the analysis of large amounts of text in a way which inductively classifies and visualises different themes and narratives (Lebart, Salem and Berry, 1998). In particular, these approaches have been applied by researchers interested in the meanings contained within political speeches (Bara, Weale and Bicquelet, 2007; Lowe et al., 2011), manifestos (Laver and Garry, 1999) and even novels (Hawthorne, 1994). As can be seen from these examples, these approaches have been used to analyse texts which can generally be characterised as long and complex. In this paper we apply the approach to much shorter and simpler open-ended survey questions.

## 5. Data and measures

We base our analyses on data from the 2010 Wellcome Trust Monitor of public knowledge, interest and engagement in biomedical science. The Monitor is a stratified, clustered probability sample, with the Postcode Address File (PAF) used as the sampling frame of households. One adult member, aged 18 or above, of each responding household was randomly selected for interview using the Kish grid procedure (Kish, 1962). The survey achieved a response rate of 59%, yielding 1,179 adults as our analytical sample size (see Butt et al., 2009 for full technical details of the survey). The strength of the Monitor for our purposes here is that, while predominantly employing standard closed-ended questions, it also contains several open-ended questions, in which respondents were asked to say, in their own words, what came to mind when they heard a particular scientific term or phrase. The interviewer transcribed the verbatim reply given by each respondent and it is these free-form text responses on which we base our analysis. Owing to restrictions of space, we focus here on a single question which asks respondents to report what comes to mind when they hear the term “DNA.” Analyses of an additional question on “stem cells,” which show a broadly similar pattern of results are reported more fully in Stoneman, Sturgis and Allum (2011). In order to avoid asking people to talk openly about something they have never heard of, respondents were first asked to rate their level of awareness of the term.^2^ Only respondents who indicated that they thought they had some understanding of DNA were then administered the following question “What do you understand by the term ‘DNA’?” Interviewers were instructed to record, verbatim, the answers respondents provided to these questions.

## 6. Analytical approach

Our analysis uses the set of statistical procedures encoded in the software package Alceste (Reinert, 1998) to cluster verbatim responses to open survey questions into groupings which reflect common underlying narrative structures. In conceptual terms, an Alceste analysis seeks to inductively reveal common narratives or discourses within a body of textual data. In the context of biomedical applications, for example, we can think of there being different ways in which individuals will conceptualise, understand and evaluate the term DNA and that this cognitive and affective variation will be reflected in the ways in which people speak about it. Thus, the procedure can be used as a means of uncovering the latent social-cognitive basis of the verbatim responses in a way that enables the structure to emerge from people’s actual talk, rather than being imposed, *a priori*, by the researcher. Alceste does not use extensive dictionaries of semantic categories for its primary analysis but, rather, it relies on the distribution of words within a body of text, often referred to as a “corpus.” Indications of meaning can be inferred by the researcher via the examination of the characteristic classes of words, which form the primary output of the program.

The Alceste program carries out a sequence of textual processing and statistical analysis procedures in order to produce its final output. The first of these is *Recognition* of dictionary words in the corpus. During this stage, a dictionary of common functional words such as prepositions, definite and indefinite articles, pronouns and so on is identified, so that these can be treated separately in the analysis. These word tokens are not used in the generation of the classes of characteristic substantive words that are the focus of the analysis. However, they are not discarded altogether but can be projected back into the classes after they have been generated, to uncover variation in the context of substantive words. For example, it would be possible to identify negative connotations of the substantive term “allowed” if “no,” “not” and “never” are identified as frequently occurring alongside this word. The next stage is referred to as *Lemmatisation*, which is the process whereby verbs and nouns are reduced to their shortest stem. This means that, for instance, “gene,” “genes,” and “genetics” would all be lemmatised to “gene+,” where the “+” indicates that more than one suffix has been detected for the common term “gene.” The purpose of this procedure is to render different versions of functionally equivalent words synonymous, in order that they are not treated as separate entities in the analysis. The automated lemmatisation procedure leaves open the possibility that some words are incorrectly allocated to a particular stem, such as “generously” to “gene+.” This was assessed manually and a small number of corrections to the default lemmatisations were identified and corrected.

The third stage is referred to as *Parsing* of the text into “context units.” With a large corpus of semi-structured text, such as focus group interview transcripts, Alceste has a procedure for parsing the text into smaller analysable chunks, which are referred to as elementary context units (ECUs). In the present case, because we have comparatively short text strings, we set the ECUs to be coterminous with the complete response for each respondent. Once these data preparation and management procedures have been implemented, a *Hierarchical descending classification* of words by ECU is carried out to produce word “classes.” A contingency table is generated from a matrix containing the ECUs in the row and the words (presence or absence of all substantive words in the corpus) as the column variables. The program begins with all of the ECUs as one class and then iteratively splits the corpus into two maximally distinct sub-classes according to a Chi-square criterion. In other words, the ECUs are first split into two groups and the group indicator is cross-tabulated with the words. This procedure is a variant of hierarchical divisive cluster analysis (Kaufman and Rousseeuw, 2005). The squared difference between the observed and the expected word frequencies is then evaluated. This process is repeated until the two maximally different classes are found in terms of the distribution of words.

In a final stage, the set of derived classes is cross-tabulated with the words in the corpus and subjected to a correspondence analysis. This is a geometric technique for visualising the variation in a contingency table in a low-dimensional space (Greenacre, 2007) and can be thought of as analogous to a principal components analysis for categorical variables. The output from this analysis can be used to identify the proximity of words and classes to each other along the key dimensions of variation.

## 7. Results

Before presenting the results of the Alceste analyses, it is useful to provide some descriptive information about the nature of the responses provided to the open-ended question which asked respondents to think about “DNA.” The proportion of individuals who did not provide any verbatim material at all was just over 10%. This figure includes those respondents who said they had not heard of DNA, so were not asked the open-ended question, along with the small number of respondents who were asked the question but did not provide any verbatim response (17 for DNA). Nonetheless, close to 90% of respondents offered some form of response, indicating that there is widespread familiarity with the term.

The most frequently used terms to describe DNA were: “gene,” “make up,” “person,” “individual,” “cell” and “unique.” This suggests that the general narrative around DNA is that it is related to genetics and it is part of what makes individuals different from one another. There are also some not so intuitive terms such as “fingerprint” and “blood” which would seem to be related to respondents linking DNA with criminal investigations. These potentially different ways of thinking about DNA can be seen in the examples below which are from the verbatim responses (the respondent’s unique serial number is in parentheses):

(111171) A unique human fingerprint

(112051) Body’s blueprint; specific to you as an individual

(133201) Double helix – the blue print of life

(141111) It’s the code that is passed on to your personality

These four examples demonstrate the idea of DNA being the basic code upon which our individuality is built, making us “who we are.” In this sense, DNA is defined in terms of *what it does*. But there are also many “non-functional” accounts of DNA offered. Compare the first and second pair of responses below:

(111231) Used to determine the true father of a child

(138011) What I have seen on police things – they take swabs out of your mouth if you’ve been to a certain place

(123121) Deoxyribonucleic acid is the formation/structure of the cell nucleus to determine the character of any living organism

(127191) Coil of atoms

The first pair focus on how our knowledge about DNA is *applied* to everyday life, with genetic parentage and criminal investigations as examples. The second pair offer *ontological* definitions of DNA, which in strict technical terms is the most accurate type of response. Nonetheless, even when offering this type of response, we can see from the two examples above that the first one is full and completely accurate while the second, whilst trying to define what DNA actually is (as opposed to what it does or how it is applied) relies on a visual representation and not a technical definition.

Of course, there are severe methodological weaknesses with relying on subjective scanning of the verbatim responses and the selection of “illustrative” quotes. First, it uses information from only a tiny fraction of the total sample and, second, there is no way of telling how “representative” these selections really are.

## 8. Alceste analysis of the DNA verbatim responses

The results of the Alceste cluster analysis showed that there are seven classes of verbatim responses. The not-asked category (11.3%) is comprised almost entirely of those who said they were not familiar with the term DNA and so were not asked the verbatim question. There is a second non-substantive group of respondents who provided some verbatim response to the open-ended question but whom it was not possible to allocate to one of the narrative classes. This group is labelled “unclassified” and comprises almost a quarter of all respondents. For those who were allocated to one of the five substantive classes, class 1 is the biggest (23.7%) followed by class 2 (15.9%), class 3 (10.6%), class 4 (8.1%) and finally class 5 (7.8%). There is a good deal of variation in the number of unique words found within each class. Class 2 is the most numerous with 69 unique words, while classes 3 and 5 have 44 and 38 terms respectively. Classes 1 and 4 are very homogeneous with only 20 and 19 unique words in them respectively.

To aid the interpretation of what the narrative structures and discourses underlying the substantive class formations are, Table 1 shows the most common words defining each class. Class 1 is defined by a discourse about genetic make-up. People understand that DNA defines individuality in some way, related to genes, that is, they focus on the direct effect of DNA on people. Representative verbatim responses within this class include “make up of a persons cells which makes each person an individual” and “your genes and cells your body is made up of.” Class 2, on the other hand, appears to be focused on the function and uses of DNA testing, such as proving paternity and solving crimes by taking samples of blood or other tissues. As such, this class seems to capture a view about DNA primarily informed by how the term commonly arises in popular culture and one that is largely absent of technical terms, evident through verbatim responses such as “its taking samples of blood or tissue to identify murder victims or fathers of children” and “blood tests that you take to identify you and to prove that you are the parent of a child.”

**Table 1.** Common words within the substantive Alceste classes.

Class 1			Class 2			Class 3			Class 4			Class 5		
24% (*n* = 280)			16% (*n* = 187)			11% (*n* = 125)			8% (*n* = 96)			8% (*n* = 92)		
Word	Freq	%	Word	Freq	%	Word	Freq	%	Word	Freq	%	Word	Freq	%
gene	222	71	find out	27	100	acid	50	96	personal	20	85	everyone	60	63
make up	206	71	parent	36	86	life	46	93	human	40	55	hair	27	85
individual	107	68	test	35	86	building block	48	90	living	28	57	DNA	123	37
make	60	75	child	27	93	helix	22	95	identify	32	53	leave	8	100
map	10	100	prove	20	95	organism	14	100	basic	9	89	saliva	26	58
body	112	52	sample	33	79	deoxyribonucleic	15	93	signature	6	100	finger	9	89
cell	76	53	identify	49	67	protein	13	92	footprint	11	73	own	44	44
pattern	13	77	take	33	76	dioxin	4	100	molecular	7	86	different	53	53
			murder	14	100	nucleus	4	100	link	4	100	skin	8	8
			blood	54	61	instruct	4	100	imprint	6	83	their	31	31
			father	24	79				unique	81	27	fingerprint	66	33

Class 3 consists of what appear to be, in many cases, a scientifically accurate description of DNA, with the vast majority of responses offering a more technical definition such as “the double helix structure carrying all the gene information colour of eyes etc” and “deoxyrybo nucleic acid the building blocks of all life.” Class 4 also contains statements that accurately describe the nature of DNA, but in contrast to class 3, there is an absence of “technical” language. These responses are couched in terms of a blueprint or a signature, such as “it is the basic molecular structure of living things a unique footprint” and “its the basic blueprint for all living things,” with little biological or technical knowledge evident. With class 5, DNA is described in terms of how it can be used to identify people and how it can be thought of as analogous to a “fingerprint,” but, importantly, a lot more uncertainty is expressed, with responses such as “cant explain everyones dna is different” and “not everyone has the same dna don t know any more” being relatively common in this class.

What we have then are broadly three sets of classes: class 2 offers a rather general description, focusing on how DNA is used in medicine and criminal investigations, while individuals within classes 3 and 4, and to a lesser extent class 1, offer a direct definition of what DNA actually is (an “ontological” definition), with class 3 demonstrating more technical knowledge than classes 1 and 4. Finally, class 5 contains individuals who only tentatively offer a definition and are quite honest that they lack confidence in what they are expressing.

**Figure 1.**
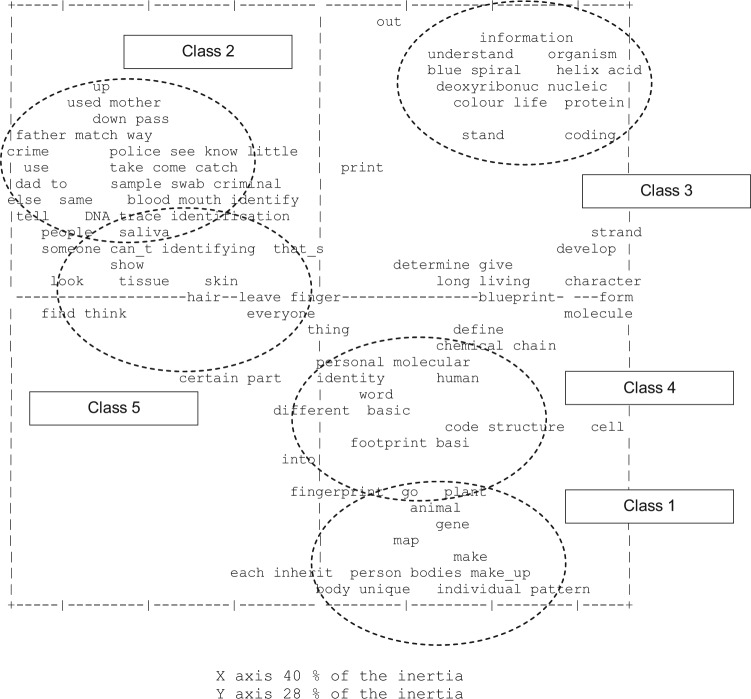
Alceste correspondence analysis: DNA.

The Alceste-generated contingency table which outlines the clusters and associated key words can be presented graphically as a correspondence plot which will identify similarities and/or differences between the classes. From Figure 1, we can see that all of the descriptive discourse is on the right hand side of the chart, while the more function-based classes appear on the left. Thus, we can think of members of the public being positioned on a dimension from description to functionality in relation to DNA. The accurate description in class 3 is the most distinctive class, in terms of its distance to the middle of both horizontal and vertical axes, and in terms of the distance between it and the other classes. Respondents in class 3 appear, then, to be the most distinct group.

## 9. Do the narrative classes have explanatory power?

Thus far we have shown that the verbatim responses can be partitioned into substantively interpretable groupings that vary with respect to how people talk about an aspect of biomedical science when asked to do so in their own terms. An important additional consideration is to what extent these derived classes possess explanatory, as well as descriptive power. The Alceste-generated classes are intended to represent *different ways of thinking* about DNA, and it is illuminating to explore whether this dimension of variation provides additional analytical leverage in explaining relevant attitudinal dimensions. We therefore used class membership dummy variables in regression models alongside a set of “standard” explanatory variables to predict an attitudinal indicator of optimism about medical science and genetic research. The wording and response alternatives for this attitude item were:

“How optimistic are you about the possibility of medical advances as a result of genetic research?” (1 = very optimistic; 2 = somewhat optimistic; 3 = not too optimistic; 4 = not at all optimistic)

The frequency distribution for this variable is presented in the Appendix. Owing to the positive skew of the density function, this variable was re-coded into binary form, where “not too optimistic” and “not at all optimistic” are coded 0 and “somewhat optimistic” and “very optimistic” are coded 1. Nested binary logistic regression models were then used to predict the probability of being “somewhat optimistic”/“very optimistic” about the medical benefits of genetic research.

Because the narrative classes appear to partially reflect differences in understanding of what DNA is and what it can be used for, it is important to evaluate whether the classes are able to account for between-person variability in optimism, over and above conventional measures of scientific knowledge (Miller, 2001; Sturgis and Allum, 2004). We also include as covariates a set of manually-coded variables which were derived from the same DNA open-ended question according to standard procedures (Groves et al., 2009). That is to say, trained coders in the survey data collection agency developed and applied thematic categories to the verbatim responses in order to transform them into nominal variables. This resulted in seven manually-coded variables indicating whether respondents (see Butt et al., 2009):

1. mentioned the terms “genes,” “genetics” or “genetic make-up”
2. mentioned DNA as determining human characteristics
3. focused on practical uses of DNA, such as “solving crimes”
4. mentioned parts of the body
5. offered vague or irrelevant answers
6. said “don’t know”
7. offered other very specific answers such as “fingerprint.”

Four models are fitted to the data: model 1 tests the unconditional main effect of class membership; model 2 introduces covariates; model 3 includes a set of interaction terms between class membership and scientific knowledge. Interaction terms are introduced to test whether the effect of class membership is moderated by scientific knowledge. That is, in addition to any direct effect of the narrative classes on optimism about genetic science, we also assess whether any effects observed vary systematically as a function of other variables in the model. The final model, model 4, introduces the manually-coded variables. Table 2 presents the results. In model 1, we can see that membership of all the narrative classes is positively associated with optimism relative to class 2 (the reference category), with the exception of the “not-asked” group. Classes 1, 3 and 4 are very strongly associated, with odds ratios of 3.1, 8.7 and 5.5 respectively. Referring back to the interpretations of these classes, we find that offering an optimistic response to this question is strongly associated with offering an ontological definition of DNA as opposed to defining it by its effects or functions. This is especially true for class 3, in which respondents offer ontological definitions which demonstrate a high degree of technical literacy. When the control variables are introduced in model 2, the pattern of results is essentially the same but the magnitude of the coefficients declines sharply. Now, a one unit increase in knowledge is associated with being 1.4 times more likely to be optimistic and class 1 is no longer a significant predictor. The strongest predictors in model 2 are classes 3 and 4, with membership of these classes increasing the odds of an optimistic response by 2.8 and 2.6 respectively. These results suggest that the class membership variables, though strongly co-linear with science knowledge, appear to capture a dimension of cognitive engagement which is distinct from it and is, as a consequence, able to add additional explanatory utility where the objective is to understand more general attitudes toward genetic (and other areas of) science.

**Table 2.** Binary logistic regression models predicting optimism about genetic science and medical advances.

	Model 1			Model 2			Model 3			Model 4		
	B	S.E.	O/R	B	S.E.	O/R	B	S.E.	O/R	B	S.E.	O/R
Constant	0.95	0.18	2.59	−0.35	0.51	0.79	1.24	0.71	3.47	0.98	0.75	2.66
Age 22–34 (ref: 18–21)				−0.69	0.45	0.50	−0.71	0.46	0.49	−0.70	0.47	0.50
Age 35–44				−0.04	0.44	0.96	−0.09	0.46	0.92	−0.05	0.46	0.95
Age 45–59				−0.34	0.42	0.71	−0.34	0.44	0.71	−0.32	0.45	0.72
Age 60 +				−0.05	0.43	0.95	−0.07	0.44	0.94	−0.01	0.44	0.99
Male (ref: female)				0.10	0.18	1.10	0.11	0.18	1.11	0.13	0.18	1.14
No qualification (ref: GCSE/A level)				−0.37	0.21	0.69	−0.41	0.21	0.66	−0.42	0.21	0.66
Degree and above				0.47	0.32	1.60	0.43	0.33	1.53	0.32	0.34	1.38
Interest in science (ref: no)				0.32	0.23	1.38	0.31	0.23	1.37	0.25	0.24	1.28
Knowledge				0.32***	0.06	1.37	0.00	0.11	1.00	0.00	0.11	1.00
*Alceste classes (ref: class 2)*												
Not-asked	−0.38	0.27	0.68	−0.11	0.29	0.90	−2.15**	0.77	0.12	−1.93*	0.81	0.15
Unclassified	0.73**	0.24	2.07	0.37	0.26	1.45	−2.28**	0.81	0.10	−2.18*	0.87	0.11
Class 1	1.13***	0.26	3.11	0.49	0.28	1.63	0.43	1.07	1.53	0.65	1.09	1.92
Class 3	2.16***	0.45	8.65	1.04*	0.48	2.83	−8.81**	3.07	0.00	−8.87**	3.20	0.00
Class 4	1.71***	0.44	5.54	0.97*	0.46	2.64	−0.58	2.05	0.56	−0.23	2.05	0.79
Class 5	0.78*	0.34	2.19	0.43	0.35	1.54	−1.23	1.41	0.29	−1.06	1.43	0.35
Not-asked*knowledge							0.44**	0.16	1.55	0.44**	0.16	1.55
Unclassified*knowledge							0.52***	0.15	1.69	0.52***	0.16	1.68
Class 1*knowledge							0.08	0.17	1.08	0.02	0.17	1.02
Class 3*knowledge							1.65**	0.53	5.21	1.68**	0.55	5.39
Class 4*knowledge							0.31	0.33	1.37	0.28	0.33	1.33
Class 5*knowledge							0.33	0.25	1.40	0.32	0.25	1.37
*Manually-coded variables (ref: other)*												
Genetics										0.71*	0.30	2.03
Parts of the body										0.00	0.26	1.00
Characteristics										0.08	0.23	1.08
Practical uses										0.23	0.30	1.26
Vague/irrelevant										−0.09	0.42	0.91
Don’t know										0.32	0.52	1.38
Nagelkerke R	.104			.191			.227			.237		
*N*	1179			1179			1179			1179		

*Notes*: Coefficients are logit coefficients; bold coefficients indicate statistical significance where * *p* ≤ .05; ** *p* ≤ .01; *** *p* ≤ .001.

Turning now to model 3, the first thing to note is that the inclusion of the interaction terms improves the fit of the model (Nagelkerke R increases from .19 to .23), so we can conclude that science knowledge does moderate the effect of the narrative classes on optimism about genetic science. There are three significant interaction terms in model 3, with classes 1, 4 and 5 unrelated to optimism at any level of knowledge. The main effect coefficients in an interaction model denote the main effect of each variable when the other variable in the interaction is at its zero point. The interaction coefficient is interpreted as the expected change in each main effect coefficient, with each unit increase in the other variable in the interaction (Friedrich, 1982). So, we can see that, at the lowest point on the knowledge scale, being in classes “DNA not-asked,” “DNA unclassified” and “DNA class 3” is associated with being less optimistic about genetic science relative to class 2. However, as knowledge increases the negative effects of membership of these classes reduces. In short, science knowledge reduces the negative effect on optimism that is associated with these narrative classes. In model 4, the inclusion of the manually-coded variables has no effect on the significance or substantive interpretation of the Alceste class coefficients. As Table 2 shows, the introduction of these variables does add some predictive power, with those offering an explanation of DNA around “genetics” twice as likely to express optimism about genetic science (odds ratio = 2.03), but overall much greater analytical leverage is achieved with the Alceste classes.

**Figure 2.**
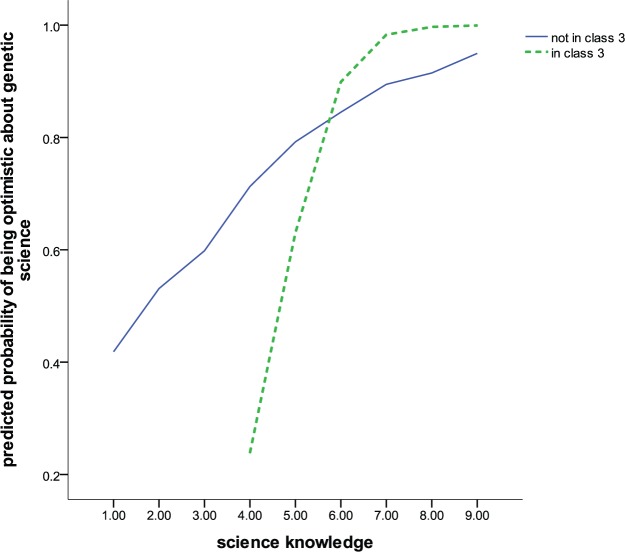
Predicted probability of optimism about genetic science by membership of narrative class 3 and science knowledge.

To aid the interpretation of statistical interactions, it is useful to produce visual plots of the model fitted values. By way of illustration, Figure 2 shows the predicted probabilities from model 4 for the interaction between science knowledge and narrative class 3. Figure 2 shows that, at low levels of science knowledge, membership of class 3 is associated with low levels of optimism, lower indeed than members of all the remaining groups combined. However, as science knowledge increases, the level of optimism increases rapidly across all respondents but particularly those in class 3, until at the highest levels of knowledge those in class 3 are the most optimistic.

Why should this be so? An advantage of the analytical approach we have adopted here is that it is possible, at this stage, to return to the full verbatim responses in order to contextualise the coefficients in the statistical model. Put differently, we are able to use the information in the verbatim responses to ascertain whether what people say at different points of the interaction can help us to understand the mechanisms that may be driving it. To this end, we collated all the verbatim responses for respondents in class 3 and compared those with knowledge scores of 5 or below to those with knowledge scores of 8 or above. This revealed that, while those in the low knowledge group still provided “ontological” statements relating to what DNA *is*, they did not appear to link this directly or indirectly to the possibility that because DNA determines cell function, understanding more about it might lead to medical advances:

(1441111) Building block of life

(1051511) It’s the mapped out life and identity at conception

(1801411) The make-up of life spiral stringy thing they discovered many years ago

These verbatim responses in narrative class 3 can be contrasted with those at the top of the knowledge scale:

(1171611) deoxyribose nucleic acid the chemicals inside the chromosomes that identify the characteristics of an individual

(1980511) it’s a double helix its basically the building block for our body works grows and develops

(1671711) deoxirebulic acid nuclear acid produces all proteins in body and determines all things like eye colour and susceptibly to diseases

In these examples, a technical definition of DNA is offered, specifically identifying it as a type of acid or mentioning the structural manifestation of double-stranded molecules of nucleic acid, the “double helix.” However, these responses also recognise that DNA plays a key role in how the human body develops and affects susceptibility to morbidity. There is some evidence in the verbatim response, then, that those in narrative class 3 who have a higher level of scientific knowledge, also have a better appreciation of genetic science’s potential for medical development and are, as a consequence, more optimistic about the potential for advances in the future.

## 10. Conclusion

It is, perhaps, something of a truism to say that quantitative researchers favour working with large-*n* datasets and testing statistical relationships between variables. Similarly, it seems almost tautological to define qualitative researchers as those who prefer the rich textual data that arise out of interviews with and observations of a small number of cases. Each tradition is, nonetheless, clearly grounded in preferences for different *kinds* of inference. The quantitative researcher strives for robust generalisation to the population as a whole and, in doing so, must make compromises over the amount (and nature) of the data that can be collected from each case. The qualitative researcher, by the same token, eschews the formalisations of population inference for the greater insight that can be gained from considering a smaller number of “illustrative” cases, often in very fine detail. In this paper we have attempted to combine the strengths of both approaches by “mixing” them within a single research design. Although interest in the idea of mixed methodological research has certainly increased substantially over the past decade and more (Bryman, 2007), it is also undoubtedly true that the goal of qualitative and quantitative *integration* is “more honoured in the breach than in the observance” (Greene, Caracelli and Graham, 1989; Niglas, 2004; Bryman, 2006). Our pursuit of methodological integration was undertaken due to long-held concerns that, for a variety of reasons, standard fixed-choice survey questions may not be well-suited to gauging public opinion about relatively novel and emerging areas of science and technology (Irwin and Wynne, 1996; Kotchetkova, Evans and Langer, 2007). It has certainly not been our intention to set fixed response alternatives and open textual data in binary opposition to one another, with the implication that one is inherently superior to the other. Rather, we have sought to explore whether undertaking a direct statistical analysis of verbatim textual responses might yield some analytical benefits when assessing public opinion in areas of science and technology which are, for many, difficult and of low salience. Our results suggest that there is some potential analytical utility for our understanding of public responses to controversial and emerging areas of science and technology by applying the rigour and inferential power of quantitative analysis to unstructured textual data collected from a large random sample of the general public.

The results of the Alceste analyses have shown that verbatim responses prompted by the term “DNA” can be classified into groups which reflect the different ways in which people think and speak about the term. That is to say, we are able to uncover latent narrative structures and discourses that are evident in the data, rather than imposed *a priori* through the production of fixed response alternatives. While the interpretation of some of the classes is somewhat ambiguous, three primary narrative discourses were evident. For some, the first thing that comes to mind when encountering the term “DNA” is how it *functions* with its ability to create “individuality” which makes us “all different” being commonly cited definitions. For others, the term evokes representations of the ways in which objects appear in everyday life and the media, with DNA anchored by narratives of its *uses*. For example, to catch criminals via “DNA fingerprinting” or identify paternity through genetic testing. A third narrative structure focused on what might be termed more “ontological” ways of thinking about DNA, with respondents attempting to provide technical “definitions” of what DNA *is* as opposed to what it does or what it can be used for.

Having demonstrated the descriptive benefits of the Alceste approach in identifying narrative discourses around DNA, a logical next step was to assess the analytical utility of the derived classes by using them as predictors of a related attitudinal dimension – optimism about genetic science – in a regression model. This showed that the Alceste classes were significant predictors of optimism about genetic science, even after controlling for standard demographic variables, scientific knowledge, and manually-produced thematic codes, which were derived using standard coding procedures from the same verbatim DNA responses. Thus, these classes appear to represent more than the degree of understanding of scientific “facts” about genetics, or the simple surface content of the responses. In addition to their main effect, we also showed that they have a strong *moderating* influence on how knowledge relates to optimism. For example, membership of narrative class 3 was associated with lower levels of optimism relative to the other classes when scientific knowledge was low. However, at higher levels of knowledge, this position switched such that membership of class 3 was associated with the highest level of optimism. A particular benefit of directly analysing the textual data was that we were able to gain additional insight into potential mechanisms underlying this interaction by re-integrating the qualitative data alongside the quantitative results.

Although these procedures offer additional insights relative to a standard quantitative analysis, two primary limitations with the approach were evident. The first of these relates to the ontological status and meaning of the derived classes. That is to say, some of the derived classes were difficult to interpret substantively, given their loose structure of associated words. This difficulty of interpreting the substantive meaning of inductively derived latent structures is not unique to Alceste (Kline, 2010). Nonetheless, it is clear that the somewhat ambiguous nature of the derived classes, despite their evident analytical power, must be considered a limiting feature of this approach, particularly if the intention is to inform policy interventions and other areas of decision-making. A second limitation is that many of the verbatim responses were very short, containing just a few words in many instances. Most often, qualitative analysis is carried out on much larger corpora of text – paragraphs, speeches, interviews, manifestos or official documents (see for example Bara et al., 2007; Schonhardt-Bailey, 2005). As we were working with, on average, just twelve words for each unit, our search to uncover latent narrative structure was necessarily constrained. This is not, of course, an inherent limitation of the method but clearly indicates the need to implement ways of collecting verbatim responses which maximise the semantic content of the data obtained. One interesting direction that might be usefully pursued in this regard would be to use audio-recording of the verbatim responses, which could later be transcribed (possibly via automated procedures), rather than using interviewers to type the oral responses manually. Although this, in turn, raises important practical and ethical issues relating to respondent consent and confidentiality, it is almost certainly the case that for the true power of quantitative textual analysis to be realised, the current means of collecting verbatim responses in surveys must catch up with the methods that have been developed for analysing them.

## Notes

1. See http://www.dailymail.co.uk/news/article-1006594/Two-people-hybrid-embryos.html

2. The question wording was, “I’d now like to ask you about your understanding of different scientific terms that are used in news stories dealing with medical research. First, when you hear the term DNA, how would you rate your understanding of what the term means: 1. I have a very good understanding of what DNA means 2. I have a good understanding 3. I have some understanding 4. I have heard the term “DNA” but have little understanding of what it means 5. I have not heard the term DNA.” Only respondents selecting response alternatives 1–3 were administered the open-ended follow-up question.
